# Comparative analysis of novel preprocessing techniques and deep learning based multi modal feature fusion for diabetic retinopathy grading

**DOI:** 10.1038/s41598-025-31339-w

**Published:** 2025-12-12

**Authors:** B. C. Anupama, Sheela N. Rao, M. Bindu Malini, V. Vikram Athreya

**Affiliations:** 1 Department of Electronics and Instrumentation, JSS Science and Technology University, Mysuru, India; 2 Department of Ophthalmology, JSS Medical College and Hospital, Mysuru, India; 3 Department of Mechanical Engineering, The National Institute of Engineering, Mysuru, India

**Keywords:** Contrast enhancement, Eye fundus images, Illumination correction pre-processing, Retinal diseases, Health care, Medical research

## Abstract

Fundus images are crucial for the detection and monitoring of retinal diseases such as diabetic retinopathy (DR). However, issues such as uneven illumination, low contrast, and noise often degrade image quality, impacting the accuracy of automated grading systems. This study introduces three novel preprocessing techniques Adaptive Sigmoid Enhancement, LAB-ACE Image Enhancement, and Multi-channel Image Enhancement designed to address these challenges. Adaptive Sigmoid Enhancement adaptively adjusts local contrast to highlight subtle lesions, LAB-ACE operates in the LAB color space to selectively enhance the lightness channel while preserving color fidelity, and Multi-channel Image Enhancement applies targeted green-channel optimization combined with contrast stretching and channel recombination. These methods extend beyond conventional contrast enhancement and normalization by integrating multi-stage adaptive processing and color-channel-specific optimization to improve lesion visibility and vessel delineation while minimizing background noise. Following pre-processing, handcrafted features (LBP, GLCM) and deep features from a pre-trained ResNet-50 are fused in a multi-modal framework and evaluated using multiple classifiers, including SVM, KNN, Random Forest, and XGBoost. Results demonstrate that XGBoost with fused features and Adaptive Sigmoid Enhancement achieves the highest accuracy (96.39%), outperforming other combinations. The findings highlight the effectiveness of the proposed pre-processing strategies in enhancing DR grading performance, paving the way for improved computer-aided diagnosis systems.

## Introduction

**Fig. 1:**
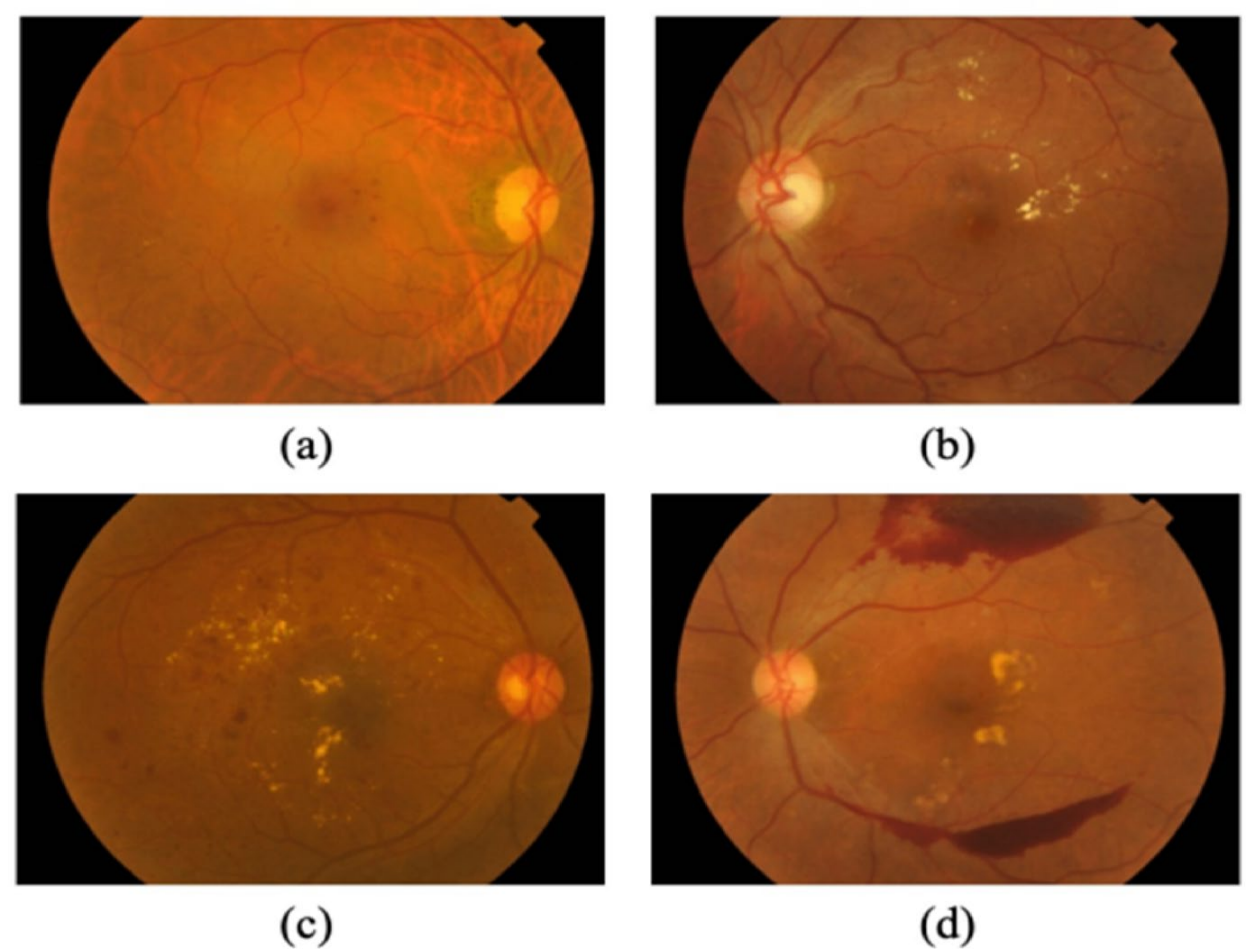
Four DR phases derived from the APTOS 2019 dataset: (**a**) mild, (**b**) moderate, (**c**) severe, and (**d**) PDR.

 The progressive microvascular problem known as diabetic retinopathy (DR), which results from diabetes mellitus (DM), damages the retina and may cause blindness. This disorder arises from chronic hyperglycemia, which results in vascular anomalies such as capillary blockage and leakage^1^. Given that diabetes is becoming more commonplace globally, early detection and treatment are essential to avoiding irreversible blindness^2^. The professional appraisal used in traditional screening techniques, such fundoscopic exams, can be laborious and subjective. Therefore, automated diagnostic methods that make use of image processing and machine learning have drawn a lot of interest in order to improve the efficiency and accuracy of screening^3^. DR develops in distinct phases according to the degree of retinal alterations. Microaneurysms, which are tiny dilatation in the retinal capillaries, are a hallmark of the first stage, mild non-proliferative diabetic retinopathy (NPDR)^4^. More haemorrhages and mild vascular anomalies result from greater microvascular damage as the disease progresses to moderate NPDR. Widespread capillary occlusion in severe NPDR increases the likelihood of disease development by causing venous beading and large haemorrhages^5^. The formation of aberrant blood vessels as a result of retinal ischaemia characterises the last stage, proliferative diabetic retinopathy (PDR), which greatly raises the risk of vitreous haemorrhage and retinal detachment, which may result in blindness^6^. Artificial intelligence (AI) and deep learning methods used to retinal imaging have advanced because to the need for accurate DR classification^7^. Figure 1 shows the different stages of fundus image from APTOS database.

**Fig. 2:**
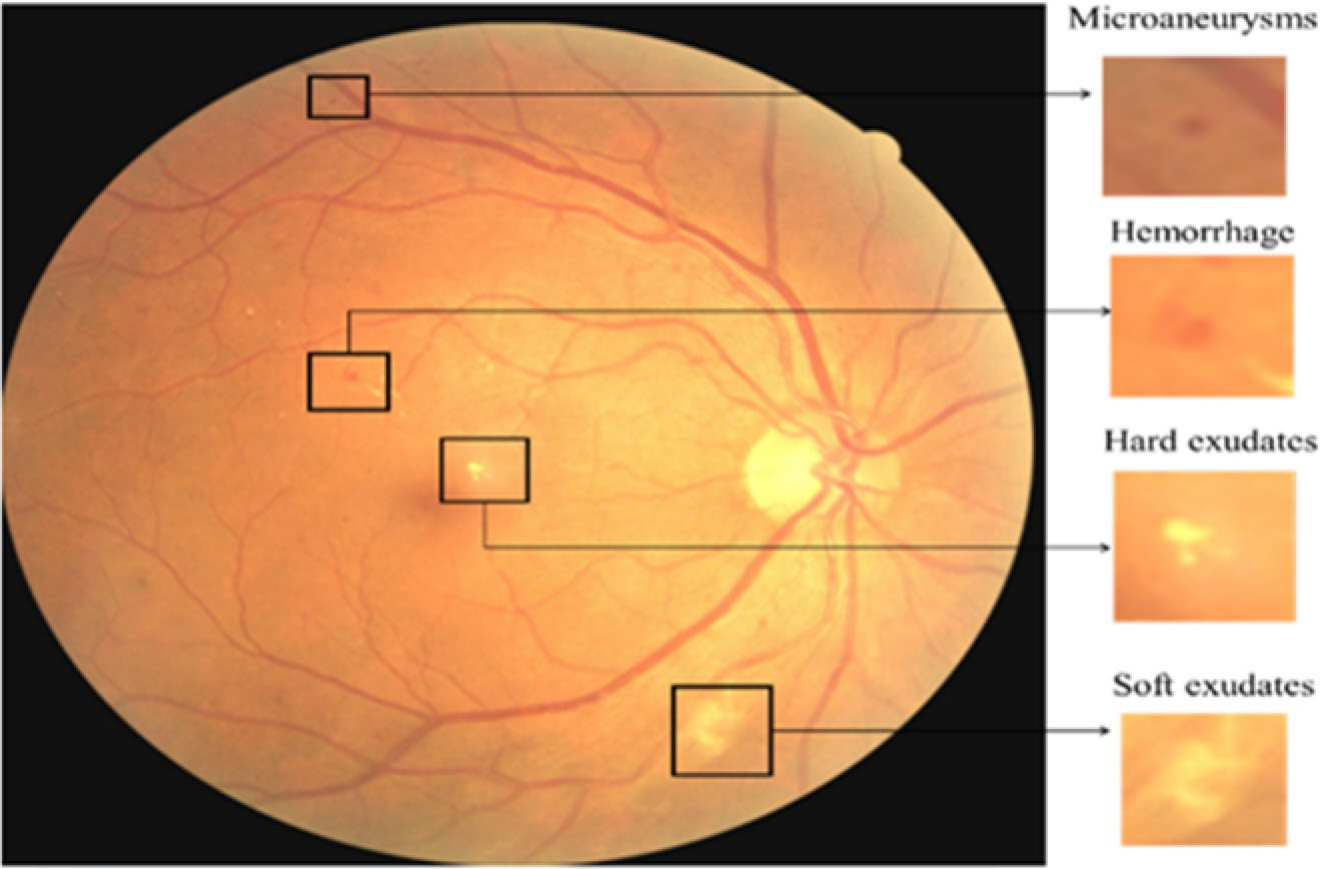
Fundus picture for DR classification showing different lesions.

Although recent studies have shown that these models have the potential to increase diagnostic accuracy, issues including inconsistent picture quality, unbalanced datasets, and the interpretability of AI-driven judgements still exist. With the goal of outperforming current classification performance while maintaining robustness and clinical application, this study proposes an improved method for DR grading employing sophisticated feature extraction and machine learning techniques in MATLAB. Figure 2 shows the various lessions present in fundus images for DR classification.

**Fig. 3:**
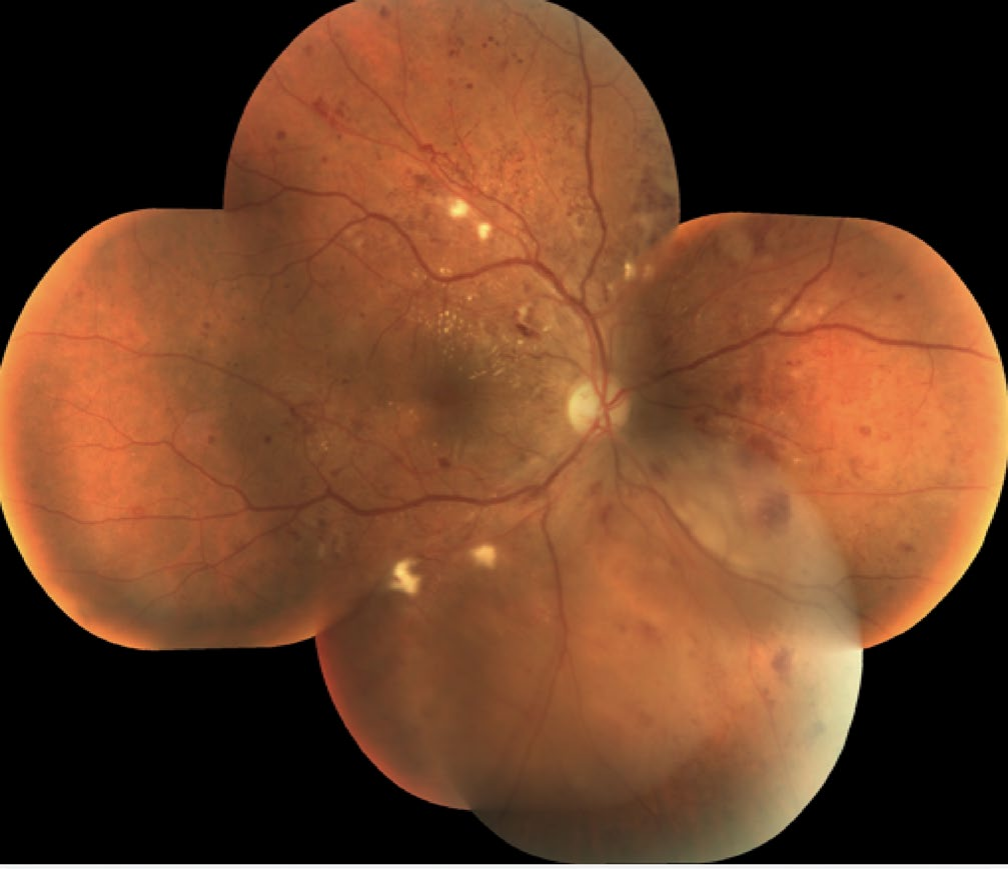
Raw fundus image of human eye.

Retinal pictures of high quality are essential for accurate DR detection and classification, in addition to strong machine learning models. Effective pre-processing methods are therefore necessary to improve image quality, lower noise, and standardise inputs, all of which will eventually boost the functionality of automated DR detection systems. Usually, imaging of the eye fundus or what is commonly known as fundoscopy or retinal photography is important for diagnosing and monitoring numerous retinal issues like diabetic retinopathy, age-related macular degeneration, and glaucoma. These photos are useful for early recognition and accurate diagnosis because they provide clinicians with information about the vascular and structural conditions of the retina, allowing prompt action to be taken to prevent vision impairment and other complications. Unfortunately, the challenges of capturing ocular fundus photographs often limit their applicability. For example, when images are affected by excessive noise, poor contrast, uneven lighting, and other artefacts, clinicians find it very difficult to extract valuable diagnostic information from the images. This poor image quality not only affects the accuracy of detecting diseases, but also makes subsequent analyses and treatment designs more challenging^8^. The Fig. 3 shows the raw fundus image of human eye.

Prior to additional analysis or interpretation, pre-processing approaches are utilised to boost the eye fundus image^9^ functionality, addressing a number of issues. This process includes several image enhancement techniques designed to minimize noise and other distortions while improving visual clarity, contrast, and overall quality. These techniques are crucial for transforming unprocessed, raw images into clear, high-quality representations suitable for computer-aided diagnosis and clinical evaluation. The growing demand in order to accurately and effectively detect retinal diseases has spurred significant advancements in image pre-processing techniques for eye fundus images in recent years. To tackle specific challenges in fundus image analysis, various pre-processing methods have been developed and proposed. These techniques encompass a wide range of strategies, each aimed at enhancing different aspects of image quality, including contrast enhancement, noise reduction, lighting adjustments, and artifact elimination. In this work, conduct a comprehensive evaluation and comparison of several pre-processing techniques designed to improve eye fundus images. By closely examining the benefits and drawbacks of different strategies, hope to offer insightful information about the most recent developments in picture pre-processing for the diagnosis of retinal diseases.

This study intends to create a strong framework that integrates both handcrafted features and deep learning representations through multi-modal feature fusion, given the difficulties involved in accurately grading diabetic retinopathy (DR). In order to increase classification performance, the study aims to improve the quality and consistency of input data by implementing innovative image pre-processing techniques.

The novelty of this work lies in its integrative design rather than in pre-processing alone. We introduce a comparative analysis of novel pre-processing pipelines tailored for fundus images, combined with a hybrid multi-modal feature fusion strategy that merges handcrafted and deep learning features. This approach is relatively unexplored in diabetic retinopathy grading and allows the system to leverage both interpretability and high-level feature abstraction. Furthermore, we provide a detailed evaluation of how pre-processing choices affect classification outcomes, thereby offering new insights into the interplay between pre-processing and model performance. To the best of our knowledge, this is the first study that systematically compares novel pre-processing pipelines while integrating handcrafted and CNN features for robust DR grading.

The primary objective of this study is to develop and evaluate an enhanced diabetic retinopathy (DR) grading framework that leverages novel pre-processing techniques in combination with a hybrid feature extraction strategy. Specifically, this work aims to: (i) investigate the role of targeted pre-processing operations in improving retinal image quality and the discriminative power of extracted features, (ii) design a multi-modal feature fusion pipeline that integrates handcrafted texture descriptors with deep convolutional neural network (CNN) features, and (iii) assess the performance of the proposed approach against established DR grading methods using publicly available benchmark datasets.

To find out how well the suggested framework works in actual DR grading situations, it is tested against a variety of classifiers. We tackle two main research questions to drive the course of this work: (i) How is the classification accuracy of diabetic retinopathy affected by the combination of handmade and deep learning features? and (ii) Which pre-processing techniques produce the most improvements in DR grading model performance? These enquiries serve as the foundation for our comparative research and are intended to provide insight into the best feature and pre-processing combinations for accurate automated DR diagnosis.

## Related work

The concise synopsis of the works suggested for various pre-processing techniques for DR severity rating. Researchers have presented a variety of methods for DR grading and detection that are founded on deep learning (DL) and machine learning (ML).

The detection system for diabetic retinopathy is described by Revathy R et al.^10^. Using the features that have been collected from retinal pictures, developing a hybrid ML model that can accurately identify and classify diabetic retinopathy is the main objective of this study. The colour space conversion to HSV from RGB of the retinal pictures is followed by the use of adaptive histogram equalisation, median filtering, and edge zero padding. The suggested hybrid model classified the retinal pictures as normal or abnormal with an accuracy of 82%. The F-measure score was 0.8028, both the recall and precision scores were 0.8116 and 0.8619, respectively. Recently, supervised machine learning frameworks have been effectively utilized for ophthalmic disease classification, demonstrating the growing role of AI in retinal diagnostics [^21^]. Additionally, advanced image processing and computer-aided diagnosis systems in ophthalmology continue to evolve, supporting automated clinical decision-making workflows [24].

SWATHI.C et al.^11^ have compared several pre-processing techniques for retinal fundus pictures concerning conditions such as glaucoma, hypertension, and diabetic retinopathy can be found in retinal fundus pictures. It is essential to pre-process the photos in order to eliminate extraneous noise and highlight significant details. The adaptive median filter was the most successful pre-processing technique among those examined, as evidenced by the results, which revealed that it had lower MSE value and the highest PSNR value. In contrast to the normal median filter, the adaptive median filter smoothed other kinds of noise while reducing impulsive noise and distortions with less data loss.

Toufique A. Soomro et al.^12^ their goal is to create a novel pre-processing module that will improve retinal fundus imaging and blood vessel segmentation, including tiny vessels. Additionally, it evaluates how the suggested pre-processing procedures affect the current vascular segmentation techniques. In order to improve contrast, this involves transforming the RGB image to a greyscale image and eliminating uneven illumination using morphological approaches or homomorphic filtering. For post processing module this applies a second-order Gaussian detector to enhance vessel coherence, followed by anisotropic diffusion filtering and a double-threshold segmentation technique to obtain the final binary vessel image. The proposed method achieves high accuracy (around 96%) and sensitivity (around 81%) on the publicly available DRIVE and STARE databases. It performs better than many existing vessel segmentation approaches. The pre-processing steps also improve the performance of other existing vessel segmentation methods when applied to their techniques. Several automated systems have also investigated structural retinal changes and lesion segmentation to improve feature extraction for ophthalmic diseases [26]. Prior research has also highlighted the benefit of combining handcrafted parameters with automated feature descriptors for retinal disease interpretation [27].

Shilpa Joshi, P. Karule et al.^8^ provides a comprehensive review of different pre-processing techniques, including filtering, morphological processing, adaptive contrast enhancement, colour space conversion, shade correction, and mask creation. The study discusses about how these methods are applied to overcome the difficulties in analysing fundus images and also covers a broad range of pre-processing methods that have been documented in the literature, including adaptive local contrast enhancement, colour normalisation, histogram equalisation, Gaussian automated mask creation, mathematical morphology, and filtering. These methods seek to enhance fundus picture quality and enable precise DR analysis and diagnosis.

Seyed Hossein Rasta et al.^9^ have used a number of lighting correction strategies, including quotient-based approaches, homomorphic filtering, and splitting methods. Additionally, contrast enhancement methods such as polynomial transformation as well as CLAHE. The effectiveness of the techniques was assessed statistically by determining the coefficients of variation (CV) in the red and green components of the images. Without sacrificing accuracy, the vascular segmentation algorithm’s sensitivity was raised by up to 5% using the CLAHE contrast enhancement technique in comparison to the original pictures. Rubina Sark et al.^13^ focuses on improving the application of image pre-processing and classification algorithms to the early diagnosis of diabetic eye disorders (DED) using retinal fundus images.

They used a variety of picture pre-processing methods, including lighting correction, contrast enhancement, blood vessel segmentation, optic disc detection, and exudate localization. Then utilised previously trained models for transfer learning (Xception, VGG16, and DenseNet121) and developed a new bespoke Convolutional Neural Network (CNN) model^14^ utilised for DED classification. The new bespoke CNN model that was trained using pre-processed images performed the best with an accuracy of 93.33% for diabetic retinopathy, 91.43% for diabetic macular edema, and 100% for glaucoma. The pre-trained VGG16 model also performed well, with 83.43%, 89.13%, and 88% accuracy for diabetic retinopathy, diabetic macular oedema, and glaucoma, respectively.

Dilip Singh Sisodia et al.^15^ In order to determine whether diabetic retinopathy is present, the primary goal of this endeavour is to pre-process and extract features from retinal fundus images. They pre-processed the photos by removing the green channel, boosting the contrast, and reducing them to a standard size because they believe that specific characteristics, like exudates, blood vessels, and microaneurysms, can be utilised to distinguish between normal and diabetic retinal images. They then extracted 14 features from the pre-processed images, such as optic distance, blood vessel area, exudate area, and Shannon entropy. From these 14 features, they selected 7 that were most relevant for detecting diabetic retinopathy and found that the most important feature was the exudate area, which had a mean difference of 1029.7. between normal and diabetic retinal images.

Vinya Vijayan et al.^16^ have focuses on reviewing the recent automated systems based on deep learning that recognise and categorise diabetic retinopathy using retinal fundus pictures. The study examined 24 methods for identifying and categorising diabetic retinopathy based on deep learning that were published between 2019 and 2022.Additionally, the study provided a list of publicly accessible fundus imaging datasets for the diagnosing diabetic retinopathy and talked about the common image pre-processing techniques used and review found that convolutional neural network-based methods, such as EfficientNet and its variants, have shown good performance in diabetic retinopathy identifying and rating. Techniques based on vision transformers are also emerging and have shown promising results.

Ramasubramanian.B et al.^17^ have discovered the best pre-processing strategy after examining and contrasting five distinct approaches for colour retinal pictures. Although the results of the morphological top hat approach were satisfactory, the noise could not be completely eliminated. The filter with the median reduces noise to a reasonable extent, but its features aren’t enhanced.

A. Salvatelli et al.^18^ developing efficient pre-processing methods to compensate for the different faults in retinal images is the study’s main objective. It is hypothesised that by combining image processing techniques such as morphological filtering, homomorphic filtering, and local contrast enhancement, they can boost retinal picture quality and provide more accurate feature segmentation, including exudates and blood vessels. The statistical analysis showed that the pre-processing techniques were the most effective, as they were able to rise the quantity of distinct modes in the picture histograms for 77% of the tested images. This indicates that these methods were able to reveal hidden classes in the original images.

Recent deep learning applications in ophthalmology were examined by Nguyen et al.^19^, who provided a thorough overview of the subject and described the changing landscape of CNN architectures, attention processes, and transformer models. Their review focused on the ways in which these contemporary methods have enhanced the diagnostic accuracy of detecting retinal diseases, such as diabetic retinopathy (DR). Explainability and integration into clinical workflows are two important deployment restrictions that the authors have addressed. These issues continue to be significant barriers to real-world adoption. In order to close the gap between AI performance and clinical trust, their findings highlight the significance of integrating interpretability-focused techniques, such as handmade features, with deep models.

Basarab et al.^20^ suggested sophisticated edge detection methods designed to improve DR diagnosis through machine learning. According to their research, classifier performance is greatly increased by fine-tuning picture pre-processing, especially edge enhancement, which increases lesion visibility. The study showed that models trained on edge-enhanced pictures were more sensitive in identifying the early stages of DR. The objectives of the current paper are in line with this, as dependable feature extraction and classification in DR grading systems are based on efficient pre-processing.

Skouta et al.^21^ made another noteworthy addition by conducting a thorough literature review with an emphasis on deep learning-based diabetic retinopathy evaluation methods. Methods were grouped in the review according to their performance on public datasets, feature processing techniques, and network type. In order to increase model robustness and clinical reliability, the study notably noted the growing interest in hybrid techniques, which combine handmade characteristics with deep neural representations. These revelations support the reasoning behind our study’s multi-modal feature fusion methodology.

Acharya et al.^22^ suggested an artificial intelligence-based method for early glaucoma identification that identified early indications of glaucoma using optical coherence tomography (OCT) images and an SVM-based classifier. The study achieved 91.7% accuracy by using structural and textural information from the layers of retinal nerve fibres. Relevant to our focus on diabetic retinopathy grading, their findings supports the adoption of AI-driven imaging techniques for early ocular illness diagnosis.

A multiobjective optimization-based method for glaucoma identification in retinal pictures was created by Subasi et al.^23^. They employed a feature selection method in conjunction with particle swarm optimisation (PSO) and support vector machines. By lowering false positives and increasing classification accuracy, our hybrid approach confirmed the significance of the best feature selection methods in the diagnosis of ocular diseases.

A multimodal biometric system that combined facial traits with fingerprint ridge and minutiae was presented by Shahina et al.^24^. Our use of handmade and deep learning features for multimodal fusion is precisely aligned with the fusion technique’s effectiveness in merging disparate data sources, despite its primary focus on human identification.

In a different study, Tistarelli et al.^25^ concentrated on reliable multimodal biometric systems that include several face and ridge aspects. A layered decision fusion architecture that maximised recognition performance was suggested by the study. These results provide more evidence in favour of our integrated feature strategy, which combines several modalities to improve illness classification accuracy.

Using convolutional neural networks (CNNs) trained on retinal images, Chai et al.^26^ suggested a deep learning-based approach for glaucoma subtype categorisation. With an F1-score of 0.89, the approach was able to differentiate between open-angle and angle-closure glaucoma. Similar to our staging approach for diabetic retinopathy, the authors stressed the importance of distinct feature representation for every disease type.

Using chest X-rays and CT images, Kumar et al.^27^ used nature-inspired algorithms—specifically, genetic algorithms and ant colony optimization—to choose the best features for COVID-19 diagnosis. The diagnostic performance was enhanced by their hybrid CNN-feature selection pipeline. Our own usage of pre-processing and feature optimisation for DR grading is supported by this idea of physiologically driven feature refinement.

Last but not least, Sharma and Khanna^28^ suggested an effective feature selection method for breast cancer classification using soft computing. Principal component analysis (PCA) and fuzzy logic were employed in their clinical decision support system to decrease dimensionality and enhance classification accuracy. This is in line with our strategy of improving classifier performance by fusing deep learning and handcrafted features.

Broader surveys on machine learning techniques emphasize analytical comparisons and current AI trends in medical diagnosis^29,30^. In addition, specialized feature extraction methods such as Histogram of Oriented Gradients (HOG) combined with artificial neural networks (ANNs) have been applied for glaucoma detection, underscoring the role of hybrid handcrafted–deep learning approaches. Collectively, these works confirm the promise of AI in medical image classification and motivate the development of enhanced preprocessing and feature fusion strategies, as proposed in this study for diabetic retinopathy grading.

In addition to diabetic retinopathy, several recent studies have explored artificial intelligence-based diagnostic frameworks for glaucoma and other ophthalmic conditions, highlighting the broader potential of hybrid and multimodal learning in retinal image analysis. Pathan et al.^31^ developed an automated ensemble framework for glaucoma detection using dynamic classifier selection on fundus images, demonstrating improved diagnostic robustness. Das et al.^32^ implemented a MobileNet-based deep learning model for glaucoma identification, achieving high efficiency suitable for clinical screening. Shankar et al.^33^ proposed a three-stage glaucoma classification system combining image empirical mode decomposition with deep learning, showing that multistage feature extraction significantly enhances diagnostic precision. Zhao et al.^34^ conducted a systematic review and meta-analysis of AI methods applied to OCT images for glaucoma detection, reinforcing the clinical relevance of automated image analysis. Huang et al.^35^ introduced a multimodal deep learning framework for glaucoma severity diagnosis, emphasizing the advantages of combining complementary image modalities. These recent works collectively support the effectiveness of multimodal enhancement and fusion strategies, which align with the objectives of the present study in improving the accuracy and generalizability of retinal disease classification. Additionally, automated glaucoma type identification illustrates the role of deep learning in broader ocular disease recognition []. OCT-based glaucoma classification methods further validate the benefit of AI for early detection of ocular disorders^30^]. A number of studies provide a foundation for artificial intelligence-based ophthalmic diagnosis, indicating the potential of AI to reduce clinical workload [31]. Deep learning-based ophthalmic disease grading continues to show strong performance across multiple retinal datasets and imaging modalities [51]. Analyses comparing machine learning architectures further support the applicability of AI methods for complex biomedical image classification [54, 55]. Broader studies confirm that deep architectures can generalize well across medical imaging domains including retinal analysis [56]. Similar methods have also been applied for glaucoma analysis using deep learning-based retinal image classification [57,58]. Recent analytical comparisons of machine learning techniques further demonstrate the relevance of feature descriptors in ophthalmic image interpretation [59,60]. In addition, several AI-based frameworks provide clinical insights for glaucoma screening and diagnosis, supporting automated ophthalmic clinical workflows [61–63]. 

Here, literature related to pre-processing for diabetic retinopathy grading and came up with these research gap. Pre-processing methods for diabetic retinopathy grading images are currently constrained by a singular focus, such as noise filter application or contrast enhancement, which does not provide an overall solution that manages the diverse and intricate nature of fundus images across varying medical imaging conditions and patient profiles and there is a need to explore new pre-processing methods. Also observed that pre-processed images are easier for algorithms to process which leads to faster and more efficient recognition and the categorisation of diabetic eye disease. Table 1 summaries the related work on Diabetic retinopathy grading.

**Table 1:** Shows the comparison of relevant research on diabetic retinopathy grading.

Authors	Year	Dataset Used	Features/Model	Pre-processing	Classifier/Approach	Accuracy (%)	F1-score
Revathy et al^10^.	2020	Custom	HSV + Textural	HSV conversion + AHE + filtering	Hybrid ML model	82.0	0.8028
Swathi C. et al.^11^	2017	Not Specified	Handcrafted	Adaptive median filtering	Comparative study	–	–
Soomro et al^12^.	2021	DRIVE, STARE	Vessel features	Morphological filtering, CLAHE	Gaussian + Anisotropic + Thresholding	~ 96.0	~ 0.81
Shilpa Joshi et al.^8^	2017	Literature review	Multiple types	Filtering, contrast enhancement	Not specified	–	–
Seyed H. Rasta et al.^9^	2015	Custom	Handcrafted	CLAHE, quotient, homomorphic	Vascular segmentation	–	+ 5% Sensitivity
Rubina Sark et al^13^.	2023	Private	Deep Learning (Xception, VGG16, Custom CNN)	Multi-step pre-processing	CNN + Transfer learning	93.33	–
Sisodia et al^15^.	2017	DIARETDB1	14 handcrafted features	Green channel + contrast enhancement	SVM	~ 84.0	–
Vinya Vijayan et al.^16^	2023	24 DL Methods Reviewed	DL (Efficient Ne, Transformer)	Pre-processing overview	DL-based detection & grading review	–	–
Ramasubramanian B et al^17^.	2016	Retinal color images	Qualitative Evaluation	Median Filter, Morphological Top Hat	Compared 5 methods partial noise removal	–	–
Salvatelli et al^18^.	2007	Not specified	Histogram Mode Analysis	Morphological + Homomorphic Filtering	Enhanced mode separation in histograms	–	–
Nguyen et al^19^.	2025	Not specified	Review of DL architectures (CNNs, Transformers)	DL-focused	Comprehensive DL trends for ophthalmology	–	–
Basarab & Ivanko^20^	2024	Not specified	ML classifiers with edge maps	Advanced edge detection filters	Proposed novel edge-based enhancement pipeline	–	–
Skouta et al.^21^	2023	EyePACS, Messidor, DDR	DL (CNNs, ResNet, EfficientNet)	Mixed (review paper)	Systematic review of DR deep learning methods	–	–
Acharya et al^22^.	2010	OCT images	Textural & structural features	OCT-specific enhancement	SVM	91.7	–
Subasi and Gursoy et al^23^.	2021	Retinal images	Selected features via PSO	Noise removal + enhancement	SVM + PSO	93.2	–
Shahina et al^24^.	2022	Face + Fingerprint images	Ridge, minutiae, facial features	Feature normalization	Multimodal fusion system	96.4	–
Tistarelli et al^25^.	2009	Multimodal biometric	Ridge + face fusion	None reported	Layered fusion architecture	94.5	–
Chai et al.^26^	2021	Retinal images	CNN-based deep features	Standard preprocessing	CNN	–	0.89
Kumar et al^27^.	2021	COVID-19 X-rays, CT	CNN + nature-inspired FS	Image normalization	CNN + GA/ACO	95.1	–
^28^Sharma and Khanna	2017	Breast cancer datasets	Fuzzy + PCA features	Feature scaling	Soft computing classifier	92.8	–

## Proposed DR grading framework and workflow

**Fig. 4:**
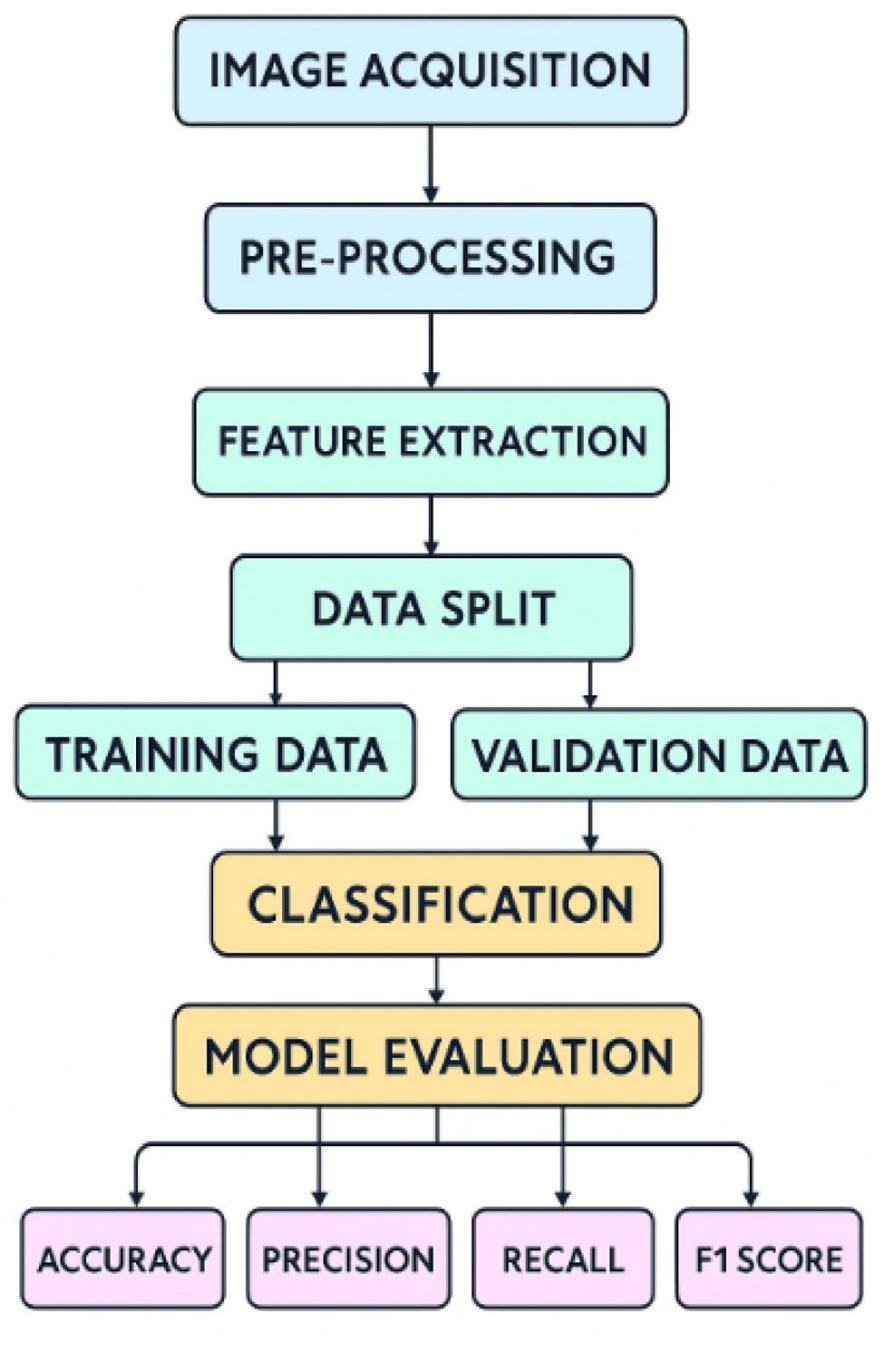
The suggested methodology’s block diagram.

The methodical pipeline of proposed diabetic retinopathy grading process, and contains data capture, pre-processing, feature extraction, data splitting, classification and model evaluation. Figure 4 displays a simple block diagram.

This work’s pre-processing, feature extraction, and classification techniques were chosen based on empirical validation as well as domain-specific expertise. Contrast Limited Adaptive Histogram Equalisation (CLAHE) was used to improve local contrast and normalise light changes, making tiny lesions like microaneurysms easier to see^36^. Because green-channel extraction has a stronger contrast for the retinal vasculature than red and blue channels, it was used because it is consistent with clinical observation procedures^37^.

Grey Level Co-occurrence Matrix (GLCM) and Local Binary Patterns (LBP) descriptors were chosen for handcrafted feature extraction because of their shown capacity to identify spatial relationships and textural patterns in retinal images^38^. Strong performance in medical image analysis tasks led to the selection of the CNN model, and cross-validation was used to adjust parameters including learning rate, optimiser, and kernel sizes in order to balance generalisation and convergence time. The proposed pipeline’s methodological transparency and repeatability are intended to be ensured by these design decisions taken together.

In this study, we employed a feature-level fusion strategy. Handcrafted features (texture, morphological, and statistical) and deep features extracted from the penultimate layer of the ResNet-50 network were concatenated into a single hybrid feature vector. This unified feature representation was then fed into the classifiers. Feature-level fusion was chosen over decision-level fusion because it allows the classifier to exploit joint relationships between handcrafted and deep features directly, thereby enabling richer discriminative modeling. This approach also reduces the need for multiple independent classifier training processes and facilitates a more compact evaluation pipeline.

### Image acquisition

Numerous retinal image databases are accessible for automated diabetic retinopathy identification and retinal image analysis. Diabetic retinopathy is graded using datasets such as IDRiD, Kaggle APTOS, and Messidor, which include several retinal images of different classes.

In this work, we make use of the APTOS 2019 dataset, which is openly accessible and comprises 3,662 fundus photos labelled with five different levels of diabetic retinopathy severity, from No DR to Proliferative DR. The detailed characteristics of this dataset, including class distribution and resolution range, are provided in Table 2.

**Table 2:** Detailed specifications of the APTOS 2019 dataset used in this study.

Dataset Name	Year	No. of Images	Classes	Resolution	Availability
APTOS 2019	2019	3,662	0: No DR, 1: Mild, 2: Moderate, 3: Severe, 4: PDR	Varies (e.g., 1024 × 1024 to 3216 × 2136)	Kaggle APTOS Challenge “https://www.kaggle.com/c/aptos2019-blindness-detection”

To provide a more comprehensive perspective, Table 3 lists additional publicly accessible retinal imaging datasets that are frequently utilised for DR detection and grading.

**Table 3:** A Comprehensive overview of publically available retinal image databases for Diabetic Retinopathy analysis..

Sl. No.	Dataset Name	Year	Number of Images	Description	URL
1	MESSIDOR1,	2008	200	Color fundus images with DR grades	http://messidor.crihan.fr
2	DIARETDB1	2007	89	Fundus images with expert-annotated lesions	https://www.it.lut.fi/project/imageret/diaretdb1/
3	DRIVE	2004	40	Digital Retinal Pictures for Extracting Vessels	https://drive.grand-challenge.org/
4	STARE	2000	400	STructured Analysis of the Retina	http://cecas.clemson.edu/~ahoover/stare/
5	EyePACS	2015	88,702	Kaggle competition dataset with 5-stage DR grading	https://www.kaggle.com/c/diabetic-retinopathy-detection
6	IDRiD	2018	516	Indian Diabetic Retinopathy Image Dataset with lesion segmentation	https://ieee-dataport.org/open-access/indian-diabetic-retinopathy-image-dataset-idrid
8	DDR	2019	13,673	Deepdr Diabetic Retinopathy Image Dataset from China	https://github.com/nkicsl/DDR-dataset
9	FGADR	2020	2,842	Fine-Grained Annotated Diabetic Retinopathy Dataset	https://github.com/csyisong/FGADR
10	ODIR-5 K	2019	5,000	Ocular Disease Intelligent Recognition (multiple eye diseases)	https://odir2019.grand-challenge.org/
11	DRISHTI-GS	2014	101	Digital Retinal Images for Optic Nerve Head Segmentation	http://cvit.iiit.ac.in/projects/DRISHTI-GS/
12	RFMiD	2021	3,200	Retinal Fundus Multi-disease Image Dataset	https://www.kaggle.com/datasets/andrewmvd/retinal-disease-classification

### Pre-processing techniques for fundus image enhancement

Image are pre-processed to enhances its features and eliminates undesired noise from the fundus image. Medical image pre-processing aims to reduce image acquisition artefacts and standardise images throughout a data collection^39^.

In this work, we use a composite preprocessing approach that combines several enhancement methods, such as bilateral filtering, gamma correction, CLAHE, green channel extraction, and sharpening. As is customary in medical image analysis, where the cumulative effect of enhancement is more therapeutically meaningful, these approaches are used in combination rather than separately. Each approach was chosen based on its proven effectiveness in enhancing fundus picture quality, as demonstrated in previous literature, even if a stand-alone ablation investigation for each preprocessing component was not carried out due to scope limitations. This guarantees that the hybrid pipelines assessed in our research are both realistically useful and supported by actual evidence. We recognise the importance of further research examining each element separately and want to pursue this avenue in order to enhance the present findings.

Unlike conventional CLAHE or global histogram normalization, the proposed methods incorporate adaptive, multi-stage enhancement tailored to retinal fundus imaging. Adaptive Sigmoid Enhancement dynamically adjusts contrast based on localized intensity profiles; LAB-ACE isolates the lightness component for targeted enhancement while preserving chromatic information; and Multi-channel Image Enhancement applies selective green-channel optimization combined with contrast stretching and channel recombination. This targeted, multi-level approach improves lesion contrast, vessel delineation, and reduces background noise, offering superior feature extraction quality compared to standard preprocessing methods.

Several pre-processing methods were explored, three methods which gave best performance has been presented here.

- Adaptive Sigmoid-Enhanced Fundus Image Pre-processing: The method consists of the following main steps:
  I. Grayscale Conversion.
  II. Adaptive Histogram Equalization.
  III. Sigmoid Enhancement.

**Fig. 5:**
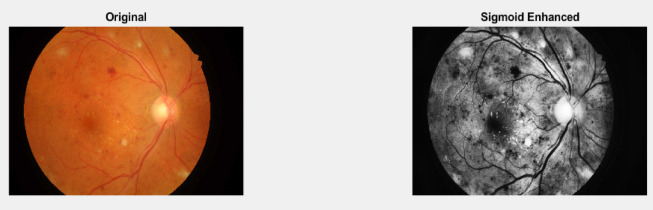
Original image and Pre-processed image using Adaptive Sigmoid enhancement.

This method uses several steps to improve fundus images. First, it balances the brightness in different parts of the image. Then, it smooths the image to remove noise. A special math function is used to make important features stand out more. The method also uses shape-based techniques to find edges and spots. All these improved parts are then combined into one image. Finally, the overall contrast is adjusted to make everything as clear as possible. This process helps make eye details easier to see, which can help doctors spot problems more easily. The Fig. 5 displays the original image and image after Pre-processing using Adaptive Sigmoid-Enhanced image pre-processing method.

b) LAB-ACE Image Enhancement: This method works in three main steps which shown below.

I. Masking.
II. Color Space Conversion.
III. Adaptive Contrast Enhancement.

The core of the preprocessing is an adaptive contrast enhancement approaches. For color images, it works in the LAB color space, enhancing only the lightness channel. For both color and grayscale images, it uses CLAHE, or Contrast Limited Adaptive Histogram Equalisation, enhances local contrast. The technique is used only to the masked region, preserving original image outside the fundus area. By enhancing contrast in the fundus region, it aims to make important features like blood vessels and potential abnormalities more visible, which can aid in medical diagnosis and analysis of eye conditions.

**Fig. 6:**
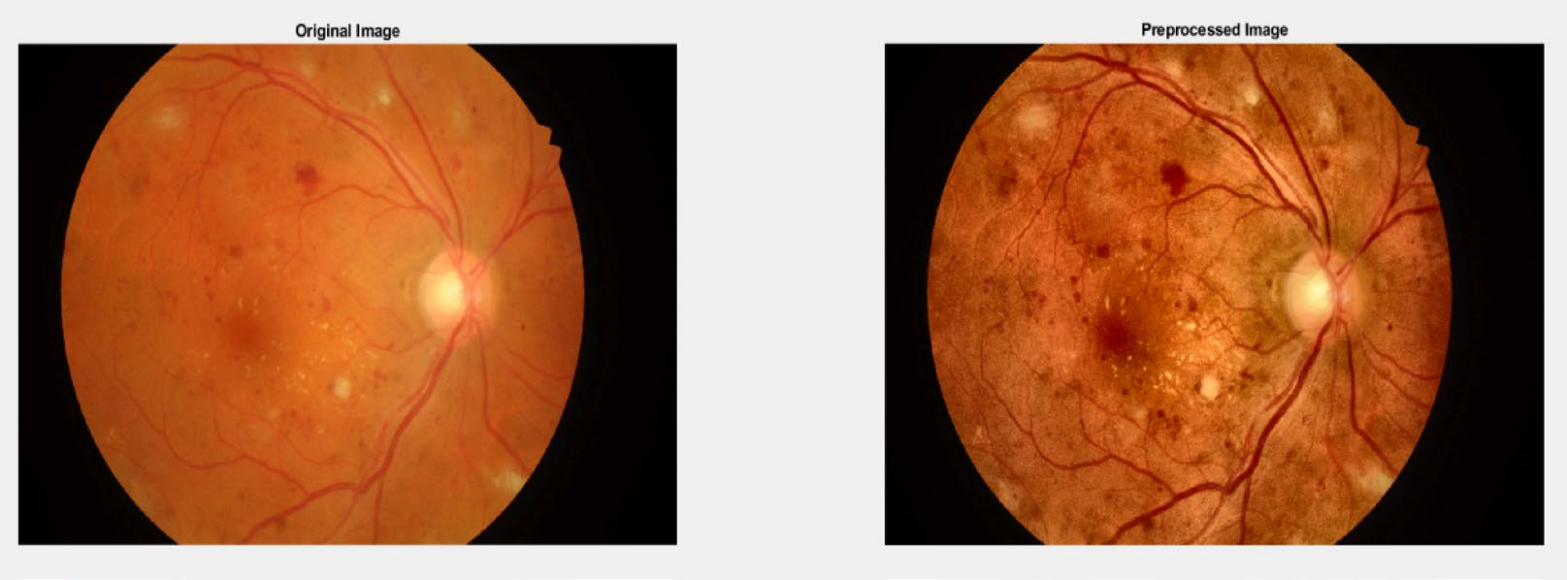
Original image and Pre-processed image using LAB-ACE image enhancement.

The Fig. 6 shows the original image and image after Pre-processing using LAB-ACE image enhancememt.

c) Multi-channel Image Enhancement: The pre-processing steps applied in this method can be described as follows.

i) Channel Separation.
ii) Green Channel Enhancement.
iii) Circular Mask Creation.
iv) Contrast Stretching.
v) Channel Recombination.
vi) Final Contrast Adjustment.

**Fig. 7:**
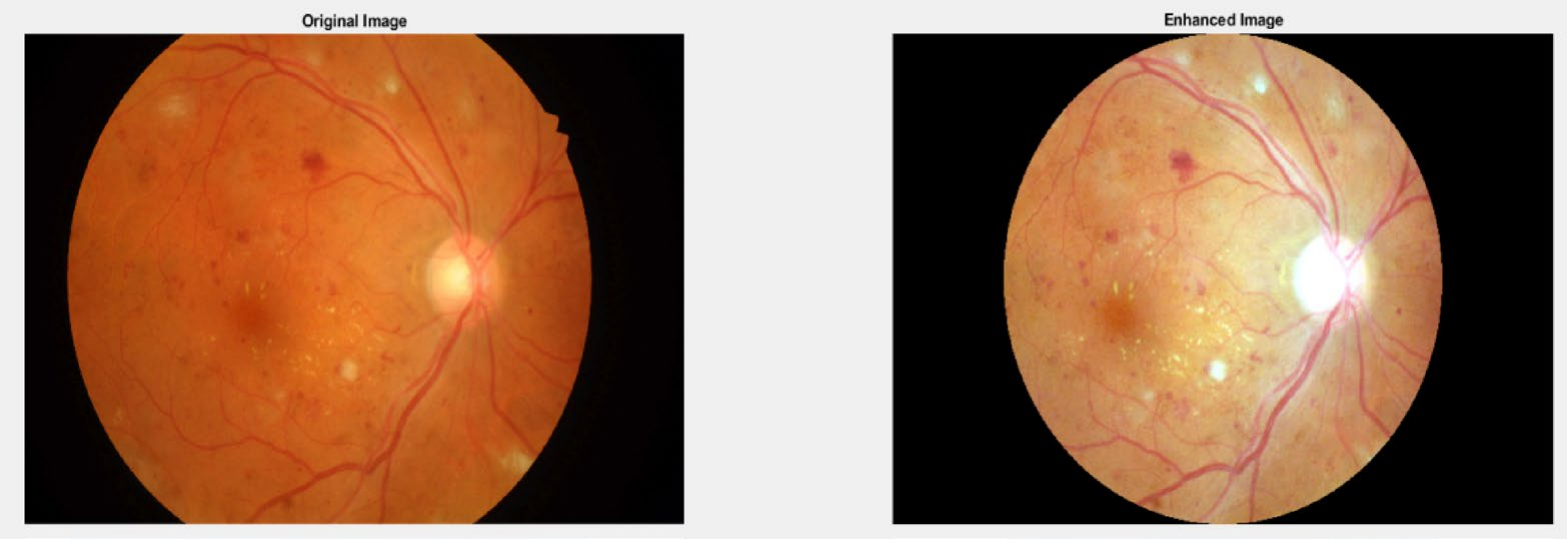
Original image and Pre-processed image using Multi-Channel Image Enhancement.

This method focuses on the green channel, which typically contains the most detail in fundus images. The process involves applying contrast stretching within a circular mask to highlight key retinal features. By enhancing color channels separately and then recombining them, it produces clearer images that reveal subtle retinal structures. This improved clarity can aid medical professionals in detecting and monitoring various eye conditions more effectively. The Fig. 7 depicts the original image and image after Pre-processing using Multi channel image enhancement.

In addition to commonly used preprocessing techniques such as CLAHE, Retinex, and homomorphic filtering, we developed and evaluated three tailored enhancement pipelines (Adaptive Sigmoid Enhancement, LAB-ACE, and Multi-Channel Image Enhancement) that focus on lesion visibility and color/luminance balance. Standard methods like CLAHE and Retinex are widely used to improve global contrast and illumination normalization, but they can sometimes amplify background noise or reduce fine lesion detail depending on parameter settings and image quality^40,41^. Homomorphic filtering is effective for illumination correction but may attenuate subtle, low-contrast lesion signals if not carefully tuned^42^. By contrast, our Adaptive Sigmoid Enhancement was designed to emphasize local contrast variations important for microaneurysms and small exudates, LAB-ACE jointly enhances chromatic and lightness channels to preserve color cues, and the Multi-channel method exploits complementary color representations to improve lesion detectability while minimizing background artifacts.

### Rationale for hybridization

The complimentary advantages of both paradigms drive the hybridisation of deep learning and handcrafted features. Microaneurysm patterns, haemorrhage borders, and exudate textures are examples of domain-specific low-level textural and structural information that can be captured by handcrafted descriptors like LBP and GLCM. On the other hand, CNN-based deep learning features encode contextual patterns and high-level abstractions that might not be specifically specified by hand-crafted rules.

Previous research in medical imaging has shown that hybrid feature sets can perform better than either purely deep learning or entirely handcrafted techniques, especially when a single representation mode may be challenged by lesion subtlety or dataset variability^43,44^. The suggested method combines both feature types in order to minimise the danger of performance degradation under various imaging situations, increase robustness to changes in picture quality, and maximise lesion detectability.

### Feature extraction

Feature extraction plays a pivotal role in medical image analysis by identifying and quantifying relevant image attributes such as texture, shape, and intensity patterns^45,46^. In this study, both handcrafted features and deep features from a pre-trained ResNet-50 network were utilized and subsequently combined in a hybrid feature fusion framework to enhance classification performance.

The handcrafted features were selected to capture complementary aspects of retinal image information and were grouped into three categories:

- Texture features:

i. Local Binary Patterns (LBP) for micro-pattern texture representation.
ii. Gray Level Co-occurrence Matrix (GLCM) descriptors including contrast, correlation, energy, and homogeneity to characterize spatial intensity relationships.
iii. Gabor filter responses for multi-scale and multi-orientation texture analysis.

- Morphological and lesion-specific features:

i. Hough transform-based detection of circular structures (e.g., microaneurysms, hemorrhages).
ii. Exudate area estimation using intensity thresholding and morphological operations.
iii. Vessel area measurement through segmentation-based morphology analysis.

- Intensity and statistical features:

i. Histogram features (mean, variance, skewness, kurtosis).
ii. Basic statistical descriptors (median, mode, standard deviation).
iii. Edge-based features extracted using Canny and Sobel operators.

For deep feature extraction, the pre-processed fundus images were passed through a pre-trained ResNet-50 model, and features from the penultimate fully connected layer were extracted. These deep features capture high-level semantic representations of the retinal images, including complex lesion structures and contextual patterns.

The goal of combining these two modalities is to improve the classifier’s capacity to discriminate between DR severity levels by utilising both clinical interpretability and representational richness. Experimental tests and previous benchmarks published in current literature were used to determine parameters such the number of epochs, learning rate, and feature vector length. Priority was given to classifiers that showed both high accuracy and stability throughout validation folds, as determined by their performance in preliminary testing. In addition to enhancing classification performance, this hybrid technique promotes transparency and reproducibility in the DR grading system.

Finally, handcrafted and deep features were concatenated to form a hybrid feature vector, enabling the classification models to leverage both domain-specific interpretable features and abstract learned representations. Parameters such as the feature vector length, learning rate, and number of epochs were optimized through cross-validation to balance accuracy and generalization.

### Classification models for DR stage prediction

 Here, several classifiers are being implemented, including XGBoost, RF, KNN, and SVM. The 3662 images will be divided into various classes by each classifier, including mild, moderate, severe, proliferative, and no DR images^47^. Various classifiers are used for dataset testing and training. A 70:30 ratios are used to prepare the training and testing sets.

### Evaluation metrics and performance comparison

The effectiveness of the various algorithms is evaluated using a number of performance analysis markers^14^. Usually, accuracy is employed as a performance indicator, representing the proportion of correctly identified images produced by the model out of all the images.

A total of 90 features that have been extracted from a data set of 3662 fundus images are given to different types of classifiers namely SVM, KNN, Random Forest, XG-Boost. The classification performance of these classifiers is evaluated by employing the confusion matrix.

A confusion matrix is a matrix that shows how well a machine learning model performs when applied to a set of test data^48^. The percentage of correct and incorrect occurrences is displayed in accordance with the model’s predictions.

The matrix displays the quantity of instances the model produced on the test data. 

True positive (TP): The actual result was positive, as the model had accurately predicted.

True negative (TN): The real outcome was negative, as the model had accurately anticipated.

False positive (FP): When the model expected a positive result, it produced a negative result instead. A Type I mistake is another name for it.

False negative (FN): When a positive result was actually obtained, the model mispredicted a negative result. A Type II mistake is another name for it.

#### Measures derived from confusion matrix information

##### Accuracy

Accuracy is used to assess the model’s performance. It is the proportion of all accurate examples to all instances.

$$Accuracy{\text{ }} = {\text{ }}(TP{\text{ }} + {\text{ }}TN){\text{ }}/{\text{ }}(TP{\text{ }} + {\text{ }}TN{\text{ }} + {\text{ }}FP{\text{ }} + {\text{ }}FN)$$

##### Precision

How well a model makes good predictions is shown by its precision. It is defined as the ratio of true positive predictions to all positive predictions made by the model.

$$\Pr ecision{\text{ }} = {\text{ }}TP{\text{ }}/{\text{ }}\left( {TP{\text{ }} + {\text{ }}FP} \right)$$

##### Recall

The ability of a classification model to recognise every pertinent instance in a dataset is measured by its recall. Itis the proportion of true positive (TP) cases to the total number of false negative (FN) and true positive (TP) cases.

$$\mathrm{Re} call{\text{ }} = {\text{ }}TP{\text{ }}/{\text{ }}\left( {TP{\text{ }} + {\text{ }}FN} \right)$$

##### F1-Score

An evaluation metric for machine learning that gauges a model’s accuracy is called the F1 score. A model’s precision and recall scores are combined.

$$F1{\text{ }} = {\text{ }}2*\left( {\Pr ecision*{\text{ }}\mathrm{Re} call} \right){\text{ }}/{\text{ }}\left( {\Pr ecision*{\text{ }}\mathrm{Re} call} \right)$$

## Results and comparative analysis

Three pre-processing methods for diabetic retinopathy (DR) grading were covered in this work. Various feature types were extracted and compared with various classifiers. The effects of each pre-processing technique on classification accuracy, precision, recall, and F1-score^49^ were used to compare their performances.

### Results before pre-processing

The Table 4 shows the results without pre-processing. Have taken the raw images directly extract the feature and given to the classifier.

**Table 4:** Results without Pre-processing.

SI. No	Features	Classification model	Precision	Recall	F1-score	Accuracy (%)
1	HOG, LBP and basic features	RF	0.6299	0.6111	0.61904	62.34
2	HOG, LBP and basic features	SVM	0.5101	0.4990	0.5023	50.55
3	HOG, LBP and basic features	KNN	0.5906	0.5701	0.5812	58.76
4	GLCM and Haralick	RF	0.5923	0.6087	0.6035	60.49
5	GLCM and Haralick	SVM	0.5012	0.4956	0.4782	46.65
6	GLCM and Haralick	KNN	0.6511	0.6710	0.6647	65.36

Based on the classification results, KNN with GLCM and Haralick features performs best overall (65.36% accuracy), Random Forest maintains stable performance independent of feature extraction method, while SVM consistently performs worse across both feature sets.

### Results of pre-processing method-1(Adaptive Sigmoid-Enhanced)

The Table 5 shows the results of Adaptive Sigmoid-Enhanced Fundus Image Pre-processing.

**Table 5:** Adaptive sigmoid-enhanced fundus image pre-processing.

SI. No	Features	Classification model	Precision	Recall	F1-score	Accuracy (%)
1	HOG, LBP and basic features	RF	0.7763	0.7612	0.7687	81.88
2	HOG, LBP and basic features	SVM	0.5322	0.5137	0.5227	55.55
3	HOG, LBP and basic features	KNN	0.7124	0.7254	0.7188	73.11
4	GLCM and Haralick	RF	0.7463	0.7389	0.7426	76.49
5	GLCM and Haralick	SVM	0.4981	0.5012	0.4996	51.65
6	GLCM and Haralick	KNN	0.6112	0.5975	0.6043	61.36

It can be observed that Random Forest with HOG, LBP, and basic features performs better than SVM models, which consistently perform worse regardless of feature set, and HOG/LBP features outperform GLCM/Haralick features in most classifiers. Random Forest also has the highest accuracy (81.88%) and F1-score (0.7687) when utilizing Adaptive Sigmoid-Enhanced pre-processing method.

### Results of pre-processing method-2 (LAB-ACE image enhancement)

The Table 6 shows the results of LAB-ACE Image Enhancement.

**Table 6:** LAB-ACE Image Enhancement.

SI. No	Features	Classification model	Precision	Recall	F1-score	Accuracy (%)
1	HOG, LBP and basic features	RF	0.7345	0.7486	0.7415	75.86
2	HOG, LBP and basic features	SVM	0.5012	0.5234	0.5120	50.70
3	HOG, LBP and basic features	KNN	0.6432	0.6546	0.6488	66.62
4	GLCM and Haralick	RF	0.7055	0.7039	0.7047	71.04
5	GLCM and Haralick	SVM	0.5097	0.5034	0.5065	51.35
6	GLCM and Haralick	KNN	0.6315	0.6274	0.6294	63.02

Results shows that Random Forest with HOG, LBP, and basic features achieved 75.86% accuracy with HOG, LBP, and basic features, exceeding SVM performance by 25.16% points across all feature sets and HOG/LBP features outperform GLCM/Haralick features in most classifiers. Random Forest also shows the highest accuracy (75.86%) and F1-score (0.7415) when using LAB-ACE Image Enhancement pre-processing methods.

### Results of pre-processing method-3 (Multi-channel image enhancement)

The Table 7 shows the results of Multi-Channel Image Enhancement.

**Table 7:** Multi-channel image enhancement.

SI. No	Features	Classification model	Precision	Recall	F1-score	Accuracy (%)
1	HOG, LBP and basic features	RF	0.7188	0.6839	0.7008	72.68
2	HOG, LBP and basic features	SVM	0.4978	0.4876	0.4926	51.60
3	HOG, LBP and basic features	KNN	0.6154	0.5964	0.6057	60.16
4	GLCM and Haralick	RF	0.6952	0.6674	0.6810	67.48
5	GLCM and Haralick	SVM	0.5055	0.4871	0.4961	49.45
6	GLCM and Haralick	KNN	0.6059	0.5987	0.6023	58.19

The classification results indicate that Random Forest with HOG, LBP, and basic features yielded the highest accuracy (72.68%) and F1-score (0.7008) among all methods tested with Multi-Channel Image Enhancement, surpassing the next-best configuration by 5.2% points in accuracy. SVM models consistently perform poorly regardless of feature set, and HOG/LBP features outperform GLCM/Haralick features.

### Results of combined hand-crafted and deep learning feature extraction for retinal image analysis

This method falls under the broader category of “multi-stream” or “multi-modal” approaches in machine learning and computer vision. It’s an example of what’s sometimes called “deep-traditional fusion” or “deep-shallow fusion” in the literature, where “deep” refers to the deep learning features and “traditional” or “shallow” refers to the hand-crafted features. These features were fed to RF classifier since it has performed best in all the above analysis. Using an RF classifier and a pre-trained ResNet50 model, deep learning features are extracted and it is displayed in Table 8.

**Table 8:** RF classifier and a pre-trained ResNet50 model are used to extract deep learning features.

SI. No	Pre-processing method	Features	Classification model	Precision	Recall	F1-score	Accuracy (%)
1	Adaptive Sigmoid-Enhancement	Custom feature + Deep learning feature	RF	0.8785	0.8990	0.8886	88.96
2	LAB-ACE Image Enhancement	Custom feature + Deep learning feature	RF	0.8276	0.8294	0.8285	83.32
3	Multi-channel Image Enhancement	Custom feature + Deep learning feature	RF	0.7865	0.8022	0.7942	79.45

It can be clearly seen that the RF classifier performs best when adaptive sigmoid-enhancement pre-processing with custom features and deep learning features are used. This results in the highest accuracy (88.96%) and F1-score (0.8886), followed by LAB-ACE Image Enhancement (83.32% accuracy), while Multi-channel Image Enhancement performs well but relatively poorly (79.45% accuracy).

### Results of Deep learning features are extracted using a pre-trained ResNet50 model and XG Boost classifier

The fusion features were also given to XGBoost classifier which is an ensemble learning classifier and Table 9 displays the outcomes of the three pre-processing techniques.

**Table 9:** Detailed specifications of the APTOS 2019 dataset.

SI. No	Pre-processing method	Features	Classification model	Precision	Recall	F1-score	Accuracy (%)
1	Adaptive Sigmoid-Enhancement	Custom feature + Deep learning feature	XGBoost	0.9456	0.9543	0.9499	96.39
2	LAB-ACE Image Enhancement	Custom feature + Deep learning feature	XGBoost	0.9023	0.9265	0.9142	91.84
3	Multi-channel Image Enhancement	Custom feature + Deep learning feature	XGBoost	0.8487	0.8712	0.8598	86.62

According to the classification results table, XGBoost performs best when Adaptive Sigmoid-Enhancement preprocessing with Custom feature + Deep learning feature combination is used. This results has highest accuracy (96.39%) and F1-score (0.9499), followed by LAB-ACE Image Enhancement (91.84% accuracy), and Multi-channel Image Enhancement (86.62% accuracy), which performs well but not as well.

Previous studies that used standard preprocessing such as CLAHE, Retinex, and Homomorphic filtering have reported performance in the range of 85–92% for DR grading^50–52^. While these approaches enhance global contrast and illumination, they sometimes amplify noise or obscure subtle lesions. In contrast, our Adaptive Sigmoid Enhancement and LAB-ACE methods specifically preserve lesion-level detail, resulting in superior visual quality and higher classification accuracy (96.39%). These findings indicate that targeted preprocessing plays a crucial role in boosting model robustness.

With the approach of pre-processing retinal images with adaptive sigmoid enhancement, have been able to achieve remarkable results with the detection of retinopathy at various stages. A total of 3,662 retinal images were added and strong classification results were achieved in all five stages of the disease. The approach has three main components, with adaptive sigmoid image pre-processing being the first, followed by custom feature extraction with a ResNet-50 deep learning model and final prediction of disease stage with an XGBoost classifier.

The superior performance of XGBoost in the experiments can be attributed to its ability to handle heterogeneous feature spaces effectively. The hybrid feature vectors in the framework, comprising both handcrafted and deep features, contain a mix of continuous, discrete, and potentially correlated variables. XGBoost’s gradient-boosted decision tree architecture excels in modeling such mixed-type data by learning non-linear relationships and performing built-in feature selection through its tree-splitting process. Additionally, its regularization parameters (L1 and L2 penalties) reduce the risk of overfitting, which is particularly beneficial when dealing with high-dimensional feature spaces derived from multi-modal sources.

**Fig. 8:**
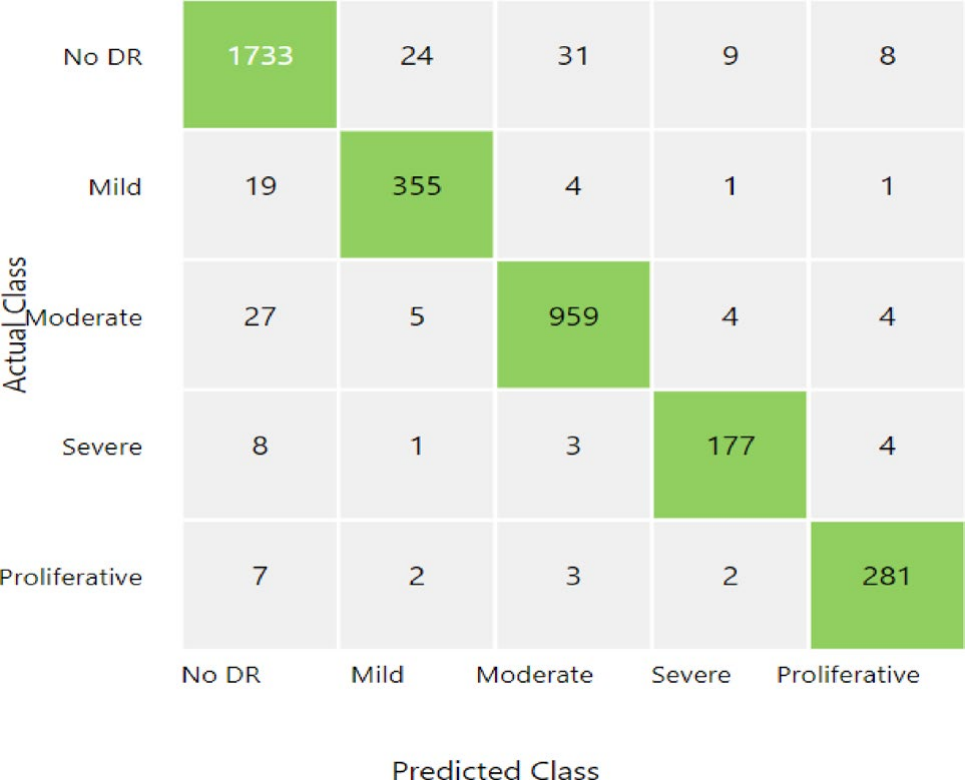
Confusion matrix for diabetic retinopathy grading using XGBoost.

Confusion matrix of DR grading with XGBoost is given in Fig. 8. The details presented on the diagonal elements of the matrix reveal that there is good predictive accuracy for all the classes in particular for the No DR cases which were correctly identified 1733 times, and 959 times in the case of Moderate DR instances.

**Fig. 9:**
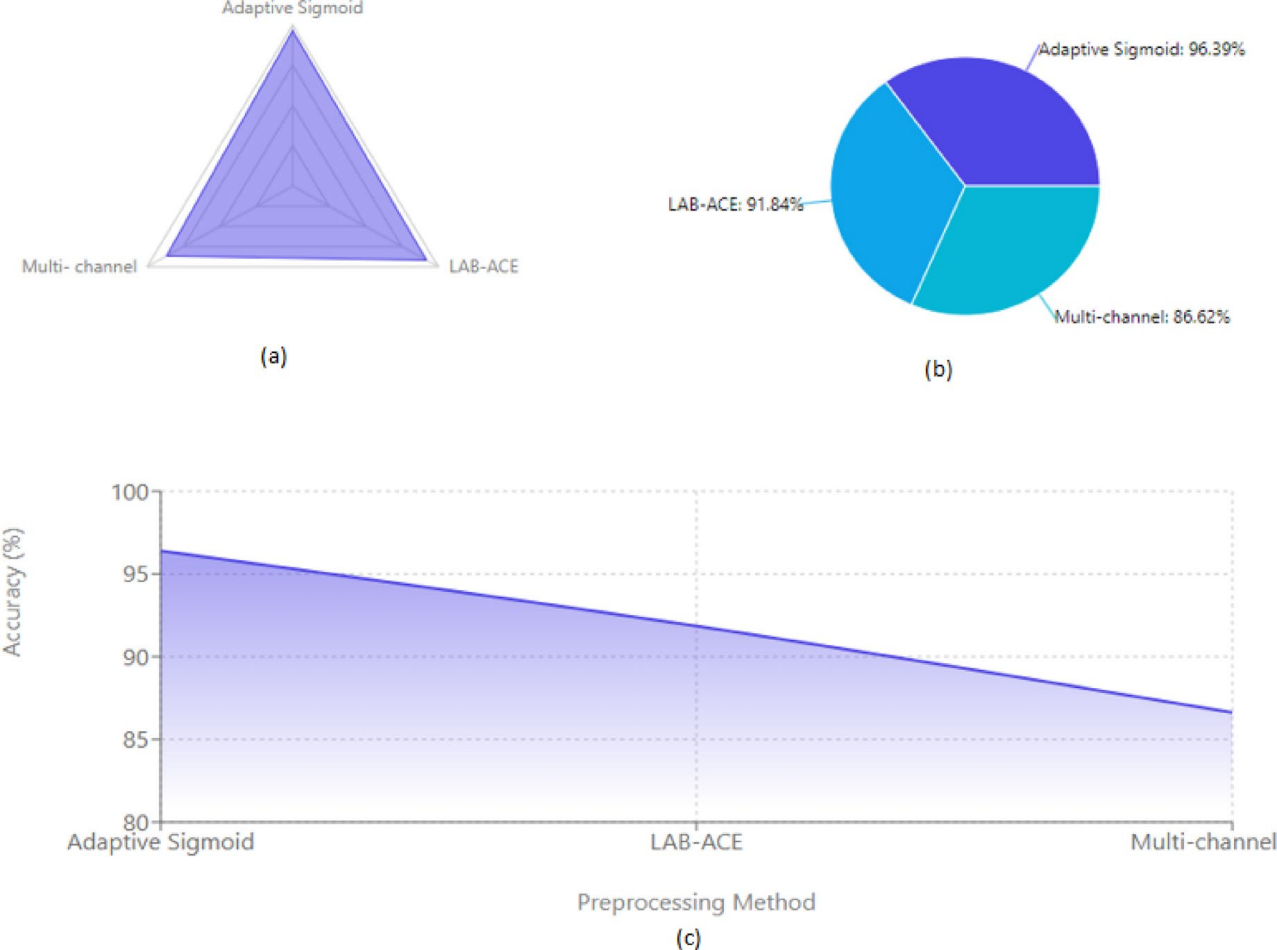
Comparison of Pre-processing Methods via (**a**) Radar chart, (**b**) Pie chart and (**c**) Area chart.

The Figs. 9 illustrate the comparative performance analysis of three pre-processing methods (Adaptive Sigmoid-Enhancement, LAB-ACE Image Enhancement, and Multi-channel Image Enhancement) with XGBoost classifier using different visualization techniques.

The Radar chart (a) demonstrates the accuracy distribution across methods, highlighting Adaptive Sigmoid-Enhancement’s superior performance at 96.39%. The Pie chart (b) presents the proportional accuracy distribution, offering a clear comparison of relative performance differences. The Area chart (c) plots the accuracy trends across methods, with the gradient-filled area emphasizing the performance gap between techniques, from Adaptive Sigmoid-Enhancement (96.39%) to LAB-ACE (91.84%) and Multi-Channel Enhancement (86.62%).

### Impact of pre-processing on classification

To assess the contribution of each pre-processing step, we conducted an ablation analysis comparing classification performance with and without individual operations. The results indicated that CLAHE alone improved classification accuracy by enhancing local contrast, thereby improving the detectability of faint microaneurysms. Green-channel extraction yielded a notable improvement in vessel segmentation quality, which in turn enhanced the discriminative capacity of both handcrafted and deep features^53^. Morphological filtering contributed to noise suppression and better isolation of lesion regions, resulting in marginal but consistent gains in sensitivity.

These observations confirm that pre-processing plays a pivotal role in shaping the quality of extracted features. Each technique contributed uniquely to the robustness of the final feature representation, and their combined application produced the highest classification performance in the proposed framework.

## Conclusion

The performance of three new pre-processing techniques was thoroughly analysed for diabetic retinopathy grading, assessing how well they performed across a range of machine learning classifiers. The experimental findings show that the adaptive sigmoid enhancement pre-processing technique produces better performance with the XGBoost classifier when paired with ResNet-50 features and custom-engineered features. This method outperformed other combinations tested in the study, achieving 96.39% accuracy and an F1-score of 0.9456. The excellent performance metrics show that our suggested approach successfully tackles the difficulties in automated diabetic retinopathy grading, especially in order to control the varying characteristics and quality of retinal images. The adaptive sigmoid enhancement method’s success can be ascribed to its capacity to improve the deep learning model’s feature extraction capabilities by normalising image contrast while maintaining important diagnostic features.

While the proposed approach shows promise, several limitations and future work directions are identified:

1. Dataset Generalization: Current evaluation is limited to the APTOS 2019 dataset. Future work should include multi-center datasets with varying imaging equipment, ethnic backgrounds, and demographic distributions to improve robustness and real-world applicability.
2. Explainable AI (XAI) Integration: The present framework does not incorporate interpretability mechanisms. Future enhancements will integrate tools such as Grad-CAM, Layer-wise Relevance Propagation (LRP), and SHAP to produce lesion-level heatmaps, improving transparency and clinician trust.
3. Real-Time Deployment: A lightweight, computationally efficient version of the framework could enable deployment on portable diagnostic devices, making DR screening more accessible in remote or resource-limited environments.
4. End-to-End Trainable Pipeline: Future research will explore integrating preprocessing, feature extraction, and classification within a single trainable architecture. Potential candidates include U-Net for lesion segmentation-driven classification and Vision Transformers for modeling long-range dependencies and global context in fundus images.
5. Multi-Task Learning: Expanding the framework to perform DR grading alongside lesion detection or severity quantification within a unified model may improve diagnostic efficiency and reduce computational redundancy. malising image contrast while maintaining important diagnostic features.
6. The proposed preprocessing and multi-modal fusion pipeline is amenable to deployment in computer-aided diagnosis systems and tele-ophthalmology platforms. Lightweight preprocessing (ASE, LAB-ACE, MIE) can be executed on edge devices or cloud microservices prior to feature extraction. Fused handcrafted and CNN features feed efficient classifiers (e.g., XGBoost) that provide fast inference and feature importance for auditability. By tuning decision thresholds for higher sensitivity, implementing a confidence-based human-in-the-loop escalation, and validating across multi-center datasets, the system can improve early lesion detection, reduce false negatives, and enable scalable remote screening workflows.

By addressing these limitations, the proposed system can evolve into a scalable, interpretable, and clinically deployable DR grading tool^54–63^.

## Data Availability

We have utilized publicly available data from Kaggle website and we have utilized APTOS 2019 from Kaggle website. Link for the dataset is provided here.“https://www.kaggle.com/c/aptos2019-blindness-detection”.

## Abbreviations

DR: Diabetic retinopathy
CNN: Convolutional neural network
ML: Machine learning
DL: Deep learning
SVM: Support vector machine
KNN: K-nearest neighbors
RF: Random forest
GLCM: Gray-level co-occurrence matrix
LBP: Local binary pattern
HOG: Histogram of oriented gradients
CLAHE: Contrast limited adaptive histogram equalization
APTOS: Asia Pacific tele-ophthalmology society
NPDR: Non-proliferative diabetic retinopathy
PDR: Proliferative diabetic retinopathy
XGBoost: Extreme gradient boosting
